# Structural homology between 11 beta-hydroxysteroid dehydrogenase and *Mycobacterium tuberculosis* Inh-A enzyme: Dehydroepiandrosterone as a potential co-adjuvant treatment in diabetes-tuberculosis comorbidity

**DOI:** 10.3389/fendo.2022.1055430

**Published:** 2023-01-09

**Authors:** Israel Hernández-Bustamante, Yanina Santander-Plantamura, Dulce Mata-Espinosa, Andrés Reyes-Chaparro, Estela I. Bini, Iván Torre-Villalvazo, Armando R. Tovar, Jorge Barrios-Payan, Brenda Marquina-Castillo, Rogelio Hernández-Pando, Andrea Carranza

**Affiliations:** ^1^ Sección de Patología Experimental, Departamento de Patología, Instituto Nacional de Ciencias Médicas y Nutrición “Salvador Zubirán”, Mexico City, Mexico; ^2^ Departamento de Farmacología, Facultad de Farmacia y Bioquímica, Universidad de Buenos Aires, Buenos Aires, Argentina; ^3^ Departamento de Toxicología, Centro de Investigación y de Estudios Avanzados del Instituto Politécnico Nacional (Cinvestav-IPN), Mexico City, Mexico; ^4^ Departamento de Fisiología de la Nutrición, Instituto Nacional de Ciencias Médicas y Nutrición “Salvador Zubirán”, Mexico City, Mexico; ^5^ Consejo Nacional de Investigaciones Científicas y Técnicas (CONICET), Buenos Aires, Argentina

**Keywords:** InhA, dehydroepiandrosterone, 11β-hydroxysteroid dehydrogenase, tuberculosis, type 2 diabetes mellitus, lung, glucocorticoid, inflammation

## Abstract

Metabolic syndrome is considered the precursor of type 2 diabetes mellitus. Tuberculosis is a leading infection that constitutes a global threat remaining a major cause of morbi-mortality in developing countries. People with type 2 diabetes mellitus are more likely to suffer from infection with *Mycobacterium tuberculosis*. For both type 2 diabetes mellitus and tuberculosis, there is pulmonary production of anti-inflammatory glucocorticoids mediated by the enzyme 11β-hydroxysteroid dehydrogenase type 1 (11β-HSD1). The adrenal hormone dehydroepiandrosterone (DHEA) counteracts the glucocorticoid effects of cytokine production due to the inhibition of 11β-HSD1. Late advanced tuberculosis has been associated with the suppression of the Th1 response, evidenced by a high ratio of cortisol/DHEA. In a murine model of metabolic syndrome, we determined whether DHEA treatment modifies the pro-inflammatory cytokines due to the inhibition of the 11β-HSD1 expression. Since macrophages express 11β-HSD1, our second goal was incubating them with DHEA and *Mycobacterium tuberculosis* to show that the microbicide effect was increased by DHEA. Enoyl-acyl carrier protein reductase (InhA) is an essential enzyme of *Mycobacterium tuberculosis* involved in the mycolic acid synthesis. Because 11β-HSD1 and InhA are members of a short-chain dehydrogenase/reductase family of enzymes, we hypothesize that DHEA could be an antagonist of InhA. Our results demonstrate that DHEA has a direct microbicide effect against *Mycobacterium tuberculosis*; this effect was supported by *in silico* docking analysis and the molecular dynamic simulation studies between DHEA and InhA. Thus, DHEA increases the production of pro-inflammatory cytokines in the lung, inactivates GC by 11β-HSD1, and inhibits mycobacterial InhA. The multiple functions of DHEA suggest that this hormone or its synthetic analogs could be an efficient co-adjuvant for tuberculosis treatment.

## 1 Introduction

Metabolic syndrome (MS) is defined as an array of metabolic disorders characterized by central adiposity, insulin resistance, dyslipidemias, inflammation, and hypertension (1) (2), which predispose patients to type 2 diabetes mellitus (T2DM) and cardiovascular diseases (3, 4). Adipose tissue inflammation is characterized by pro-inflammatory cytokines such as IL-1β, TNF-α, and IL-6 (5, 6) that are released into peripheral blood. This release creates a chronic low-grade pro-inflammatory state that affects other organs and tissues such as muscle, kidneys, the liver, and lungs (7, 8). Rodents fed a diets high in carbohydrates and fats, named as Western diets, is an experimental model of insulin resistance, impaired glucose tolerance, fatty liver, inflammation, and dysregulated immune function (9).

Glucocorticoid (GC) excess predisposes patients to metabolic unbalance that contributes to the development of T2DM. GCs are anti-inflammatory hormones secreted by the adrenal gland cortex. Intracellular levels of active GC are regulated by the enzyme 11β-hydroxysteroid dehydrogenase (11β-HSD) in peripheral tissues (10, 11). Of the two isoforms of 11β-HSD, type 1 is predominantly expressed in the liver, lungs, and adipose tissue. Type 2 is restricted to tissues such as kidneys that express mineralocorticoid receptors (12-14). The intracellular concentration of GC is regulated by the availability of the cofactor NADPH in the lumen of the smooth endoplasmic reticulum where 11β-hydroxysteroid dehydrogenase type 1 (11β-HSD1) functions as reductase by catalyzing the reduction of inactive 11 β-keto GCs (cortisone in humans and 11-dehydrocorticosterone in rodents) to active 11β-hydroxylated GCs (cortisol in humans and corticosterone in rodents). This activation increases the local action of GC (15, 16). In an experimental model of MS, intake of fructose in rodents activates corticosterone in adipose tissue through increments of 11β-HSD1 expression/activity (17). Due to its ability to increases the intracellular GC concentration, 11β-HSD1 has been proposed as a target for MS and obesity therapy (18, 19). 11β-HSD1 expression in a variety of tissues, including the lungs is up-regulated by pro-inflammatory cytokines and GCs. The inflammation in mice adipose tissue induced by a high-fat diet is a consequence of the GCs interacting with the CCAAT/enhancer binding protein (C/EBP) transcription factor, which regulates macrophage infiltration in adipose tissue (20). In the fetal lung cell line (HF-1), the inhibition of the promotor region of C/EBP down-regulates the 11β-HSD1 expression stimulated by GC and cytokines. This suggests that the up-regulation of 11β-HSD1 expression by GC and cytokines could be an auto-regulatory mechanism for lung inflammation (21, 22). There is a possibility that infiltration of inflammatory macrophages in the lungs induced by high-fat diet and fructose intake could enhance 11β-HSD1 expression and activity, which increases intracellular corticosterone availability in the lungs with the finality to overcome cytokine tissue damage. In fact, male mice fed with hyper-caloric diets developed allergic pulmonary inflammation (23). Dehydroepiandrosterone (DHEA and sulfate ester S-DHEA), the precursor of sex hormones, is produced by the adrenal cortex. Its release decreases further into adulthood. DHEA exerts anti-GC action in adipocytes (24) through the interaction with 11β-HSD1 catalyzing the interconversion of the C7-ketone to C7-hydroxyl (25).

Tuberculosis (TB) is a chronic lung disease caused by *Mycobacterium tuberculosis* (Mtb). TB causes over 1.5 million human deaths with over 10 million people suffering active disease each year, generally in middle and low-income countries (26). T2DM increases the susceptibility of developing active TB with concomitants higher rates of treatment failure and death (27, 28). In healthy human lungs, the enzyme 11β-HSD1 operates as a dehydrogenase by oxidizing cortisol to biologically inactive cortisone. In the lungs of TB patients, the cytokine over-production changes the direction of 11β-HSD1 to oxo-reductase converting cortisone to active cortisol. This change contributes to simultaneously suppressing inflammation and the protective Th1 immunity favoring the progression of the disease (29). Even more, the activation of cortisol *via* enzyme 11β-HSD1 inhibits the inflammatory process required for granuloma formation in response to Mtb, which compromises the immune control of the infection (30). The altered cytokine expression and the more severe pulmonary pathologic abnormalities in response to Mtb infection correlate with an increased cortisol/DHEA ratio in patients with T2DM and TB (31, 32). This increased ratio reduces macrophages’ activation and Th-1 response and synergizes with some functions of Th-2 response (33). Because DHEA opposes several effects of GCs *in vivo*, the increased cortisol/DHEA ratio detected in TB patients may explain the lack of an inflammatory Th1 cytokine profile with the macrophage activation necessary for the control of bacterial burden (34).

The proteins belonging to the short-chain dehydrogenase/reductase (SDR) family encompass enzymes from mammals to bacteria that have steroids, fatty acids, and alcohols, among others as substrates. Along with sharing 15–30% of their amino acid sequence identity, these enzymes share a three-dimensional structure, namely, “Rossmann fold”, in which the characteristic signature is a conserved tyrosine residue in the catalytic pocket of the active site (Tyr-XXX-Lys) and a core α/β dinucleotide binding motif (35). Mammalian enzyme 11β-HSD and the *mycobacterium* enzyme 2-trans-enoyl-acyl carrier protein (ACP) reductase or InhA (EC number 1.3.1.9) are members of this family of proteins (36). The enzyme InhA is one of the essential FASII mycolic acid biosynthesis pathway. Because mycolic acid is a component of the bacterial cell wall, InhA is one of the principal targets for new drugs development in TB. Isoniazid (INH) and ethionamide are among the most effective anti-TB drugs targeted to InhA (37). As several bacterial strains have developed resistance against the first or second line of the currently used antibiotics, there is a critical need to synthesize new molecules that may complement the currently available anti-tubercular drugs. As 11β-HSD1 and InhA are members of the SDR family of proteins, and DHEA occupies the same active site of corticosterone in 11β-HSD1, we hypothesize that DHEA could be an effective inhibitor of InhA. In the present study, we demonstrated that DHEA treatment in mice overcomes some metabolic abnormalities induced by a high-fat diet, increases the pro-inflammatory profile the in the lungs, and decreases the expression of 11β-HSD1. Incubations of Mtb with DHEA, in the presence or absence of macrophages, demonstrated a microbicide effect of DHEA. The microbicide effect could be explained through *in silico* analysis by docking simulations and the molecular dynamic between InhA and DHEA as a ligand. InhA is a critical enzyme in the synthesis of the Mtb cell wall. This is the first report about DHEA and InhA interactions that can lead to the rational design of the molecules-based hormone DHEA structure which could be an efficient co-adjuvant therapy for TB treatment.

## 2 Materials and methods

### 2.1 Animals and treatments

All procedures were carried out following “International Guiding Principles for Biomedical Research Involving Animals 2012” performed by the International Council for Laboratory Animal Science (CIOMS-ICLAS). The protocol was approved by the Institutional Council for Care and Use of Experimental Animals (CICUAL), School of Pharmacy and Biochemistry, University of Buenos Aries. For the experiment, 36 mice (C57BL/6j strain) that were 21 days old and weighed between 17 and 22 g were acclimated under a 12h light/dark period and a controlled temperature of 18–21°C. The animals were randomly divided into four experimental groups (nine mice per group). Two groups received the balanced standard diet. The remaining two groups received a fat-diet (HFD) based on AIN-93, with 45% of the caloric intake from fat (38), and unrestricted access to 10% fructose (w/v) in drinking water (39, 17) for 40 weeks. At week 28 DHEA (Cat # 704599 Sigma-Aldrich, St. Louis, MO, USA) was added during the manufacturing process (0.05% w/w) (40) to one control group and HFD diet group. The experimental groups were control diet (C), control diet plus DHEA (DHEA), high-fat diet and fructose (HFD), high-fat diet, and fructose plus DHEA (HFD+DHEA). At the end of the treatment, the animals were fasting for 12h, and anesthetized with ketamine (50 mg/kg) and xylazine (1 mg/kg); the blood was collected through cardiac puncture with a heparinized syringe before euthanizing them.

### 2.2 Tissue collection

All white adipose tissue (epididymal, visceral, subcutaneous, and retroperitoneal fat) was removed and assessed to determine the adiposity index (% adiposity = sum of the weight of all adipose tissues/total weight). Both lungs were removed and stored at −80°C. Livers were fixed in 4% formaldehyde (10% formalin (w/v) in phosphate buffer saline).

### 2.3 Plasma analyses

After centrifugation of the blood (3500*g* at 4°C for 10 min), plasma was obtained. Glycemia was measured using the Accu-Chek glucometer (Roche, Basel, Switzerland). Plasma triglycerides were determined by means of kit (Color GPO/PAP AA). Total cholesterol was determined using a kits (Colestat enzymatic AA liquid line and HDL Cholesterol monophase AA plus). Both kits were from the Wiener Labs (Rosario, Santa Fe, Argentina). Triglycerides and cholesterol measured with spectrophotometry (Metrolab 325 bd, spectrophotometer UV-Vis, Argentina). Insulin concentration was determined using the insulin ELISA kit for mice (Crystal Chem, Elk Grove Village, IL, USA ^®^) and measured at 450/630 nm in a spectrophotometer.

### 2.4 Oral glucose tolerance test

At the end of the treatment and after 12h of fasting, glycemia was measured with an Accu-Check glucometer (Roche, Basel, Switzerland). Blood was obtained from the tail vein 0, 30, and 120 min after an oral administration of glucose load (2 g/kg) by gavage.

### 2.5 Histological analysis

Fixed livers were embedded in paraffin and, vertical sections (4 µm) were cut, mounted on a glass slide, stained with hematoxylin and eosin, and observed using a microscope (Nikon Instruments, Melville, NY, USA) coupled to Micrometrics SE Premium software. To evaluate the hepatosteatosis, the livers were stained with Hematoxylin and Eosin (H&E) and scored using the NAFLD activity score (NAS).

### 2.6 Western blot of cytokines in lung

The lung tissue was homogenized in 1 vol (w/v) of ice-cold homogenization buffer (150 mM NaCl, 50 mM Trizma-HCl with a pH of 8.0 and 1% (v/v) sodium deoxycholate, 1 mM EGTA, 1 mM NaF, 1mM phenylmethanesulfonylfluoride, and 1mM sodium pervanadate) and a 4% protease inhibitor cocktail (Roche, Hertfordshire, UK). Samples were centrifuged at 10,000*g* for 10 min. Supernatants were suspended in 2X SDS sample buffer solution (62.5 mM tris-HCl buffer with a pH of 6.8 containing 2% (v/v) SDS, 25% (w/v) glycerol, 5% (v/v) β-mercaptoethanol and 0.01% (w/v) bromophenol blue), and heated at 95°C for 5 min. Proteins were quantified by the Lowry method and samples (60 μg/well) were loaded into the 10% polyacrylamide gel in SDS-PAGE buffer for electrophoresis separation, and transferred to PVDF membranes. After a 1h blockade with 5% (w/v) skim milk, the membranes were incubated overnight at 4°C with their corresponding primary antibodies (1:1000 in PBS-tween buffer): 11β HSD 1 (SC-20175), TNFα (SC-1351), IL-6 (SC-57315), GR (SC-1004), tubulin (SC-5086). They were obtained from Santa Cruz Biotech, Dallas, TX, USA. The corresponding membranes were incubated for 90 min with their corresponding HRP secondary antibodies: rabbit anti-goat IgG-HRP (SC-2768), mouse anti-rabbit IgG-HRP (SC-2357), and goat anti-mouse IgG- HRP (SC-2005). These were also obtained from Santa Cruz Biotech, Dallas, TX, USA) and kept at a concentration of 1:5000 in PBS-Tween buffer. The complexes were visualized through chemiluminescence (Pierce ECL, Thermo Fisher Scientific, Waltham, MA, USA). The densitometry analysis of the bands was performed using Image J Software (NIH, Bethesda, MD, USA). Protein band densities were normalized with respect to β-tubulin.

### 2.7 Macrophages cell line

Monocyte leukemia cell line (THP-1) was grown in medium RPMI-1640 (Gibco BRL, Grand Island, NY, USA) supplemented with 10% of inactivated fetal bovine serum and kanamycin (60 μg/ml, Gibco BRL, Grand Island, NY, USA) at 37° C in 5% CO2. THP-1 cells (10,000/well) were cultured in complete RPMI-1640 (Gibco BRL, Grand Island, NY, USA) in the presence of 30 ng/ml phorbol-12-myristate-13-acetate (PMA, Sigma-Aldrich, St. Louis, MO, USA Chemical Co.) until differentiation to macrophages. Forty-eight hours before infection, complete RPMI-1640 (Gibco BRL, Grand Island, NY, USA) was added.

### 2.8 Mycobacterium *Mycobacterium tuberculosis* strain

Studies of *in vitro* anti-TB activity were performed using the reference *Mycobacterium tuberculosis* strain H37Rv (ATCC 25618), which was incubated in the Middlebrook 7H9 broth (Difco Laboratories) culture medium supplemented with OADC (Difco ™, Thermo Fisher Scientific, Waltham, MA, USA) for 48h. Bacteria were transferred to bottles containing 60 ml of RPMI-1640 (Gibco BRL, Grand Island, NY, USA) and incubated at 70 rpm, and 35°C for 7–10 days until they reached a 600 nm OD. Mycobacterial clumps were disrupted with an RPMI 1640 medium (Gibco BRL, Grand Island, NY, USA) in 6% glycerol and vortexed for 5 min with five sterile glass beads/3ml. The suspension of bacilli was centrifuged at 900*g* for 10 min. The mean concentration of Mtb H37Rv stock was determined by counting colony-forming units (CFU) on Middlebrook 7H10 agar (Difco™, Thermo Fisher Scientific, Waltham, MA, USA) in serial dilutions using triplicates (title: 140 × 10^6^/ml).

### 2.9 Macrophages infection with *Mycobacterium tuberculosis* and incubation with DHEA

To evaluate the effect of DHEA on the phagocytic capacity of macrophages, THP-1 cells were incubated with Mtb H37Rv at a 5:1 multiplicity of infection (MOI) in the presence or absence of DHEA (0–100 μM, Sigma-Aldrich, St. Louis, MO, USA Chemical Co.) in RPMI-1640 (Gibco BRL, Grand Island, NY, USA) with 10% heat-inactivated fetal bovine serum and kanamycin (60 μg/ml, Gibco BRL, Grand Island, NY, USA) for 1h at 37°C in 5% CO2. Cells were washed three times with fresh RPMI-1640 (Gibco BRL, Grand Island, NY, USA) and then incubated at 1, 24, and 48h at 37°C in 5% CO2. Cells were lysed with lysis solution (0.1M Tris with a pH 7.6 containing 0.05% [w/v] SDS in water). CFUs were quantified by plating 10-fold serial dilutions onto Middlebrook 7H10 agar media (Difco™, Thermo Fisher Scientific, Waltham, MA, USA) supplemented with Middlebrook OADC growth supplement (Difco™, Thermo Fisher Scientific, Waltham, MA, USA). CFUs were counted after 2–3 weeks of incubation at 37°C in 5% CO2. The results are expressed as the mean of three independent experiments with two replicates per treatment (41).

### 2.10 Minimum inhibitory concentration determination

Bacterial cells were plated to achieve a final inoculum of 2.5 × 10^5^ cells per well in a 96-well cell culture dish with Middlebrook 7H9 (Difco™, Thermo Fisher Scientific, Waltham, MA, USA) 7H9-OADC-supplemented growth media (Difco™, Thermo Fisher Scientific, Waltham, MA, USA). Ten μl (per well) of each dilution of DHEA (0-500 μM, Sigma-Aldrich, St. Louis, MO, USA Chemical Co.) in DMSO were added. A row with only DMSO was also added as control of the vehicle. Plates were incubated in a humidified incubator at 70 rpm, and 37°C in 5% CO2 for 24, 48, and 72h. The CFUs were determined by plating 10-fold serial dilutions onto Middlebrook 7H10 agar media (Difco™, Thermo Fisher Scientific, Waltham, MA, USA) supplemented with Middlebrook OADC growth supplement (Difco TM, Thermo Fisher Scientific, Waltham, MA, USA). CFUs were counted after 2–3 weeks of incubation at 37°C in 5% CO2. MIC was determined as the lowest concentration of DHEA that completely inhibited visible growth (42). Three experiments with two replicates per treatment were performed.

### 2.11 Bioinformatics analysis

#### 2.11.1 Docking analysis

Because the aim was to evaluate the molecular target for DHEA, the structure of the enzyme 11β-HSD1 co-crystallized with corticosterone and the cofactor NADPH was downloaded from Protein Data Bank (PBD ID 1Y5R). The DHEA structure was obtained from the PubChem database. Energy was minimized using Avogadro software until a stable conformation was obtained (43). The enzyme was stripped from co-crystallized molecules, except the cofactor NADPH. Geometry optimization and energy minimization of 11β-HSD1were performed using UCSF Chimera with Dock Prep Tool (44). Autodock Vina software was implemented for rigid docking analysis, which demands both the ligand and the receptor in the pdbqt format (45). The pre-processing steps for ligand, protein files were performed using the Autodock Tools program (ADT). In the program, polar hydrogen atoms and Kollman charges added to the protein. For docked ligands, non-polar hydrogens were also added. Gasteiger charges were assigned and torsion degrees of freedom were located by ADT program. The Lamarckian genetic algorithm (LGA) was applied to model the interaction pattern between the receptor and the ligand (46). The amino acids of the active site (Ser170, Gln177, and Tyr183) are part of the catalytic triad found in the enzyme; they guided the location of the grid box that has the dimension of 40 × 40 × 40 (*x* = 78.495, *y* = 53.375, *z* = 34.087). Although docking predicts the strength and binding mode of a ligand, it is a theoretical simulation that needs confirmation with an experimental reference. For this reason, and in order to confirm that DHEA could interact with the active site of the enzyme, DHEA was re-docked into the active site of the 11β-HSD1 co-crystallized with its natural substrate corticosterone and the cofactor NADPH. Discovery Studio 4.5 and Chimera 1.14 visualizers were used in the analysis of docking results (44).

In order to evaluate the ligand capacity of DHEA, the crystal structure of enzyme InhA (complexed with NADH was downloaded from the Protein Data Bank (ID: 4TRN) and ligand DHEA from the PubChem database. Docking simulation was performed as described above. The amino acids of the active site (Lys165, Thr196, and Tyr 158) are part of the catalytic triad found in InhA; they guided the location of the grid box. The grid box was determined with a size 30 × 26 × 27, which surrounds the binding domain of the co-crystallized ligand with the enzyme (*x* = 8.58, y = −32.87, and *z* = 13.9). The grid box is large enough to include the NADH, as well as the substrate-binding cavity of InhA.

#### 2.11.2 Molecular dynamics

Molecular dynamics tests were carried out to evaluate the stability of the ligand-receptor complexes that resulted from the molecular docking tests. The charmm-gui platform was used to prepare inputs. The Gromacs 2021.1 software was used for molecular dynamics (47). Each protein was preprocessed using the PDB reader tool (48). The resulting docking ligands with the highest affinity energy were selected and changed to mol2 format using OpenBabel (49). The mol2 files of the ligands were loaded into the Ligand Reader & Modeler tool to generate the parameters and topology files (50). The ligand-receptor complexes were integrated into a single pdb file to be used in the “Solution Builder” tool. The tool was used to create the system that was used for the Gromacs input (51). The water box was cubic, fit to protein size, and had 10 Å of edge distance. Each system was neutralized using KCl ions placed by the Monte Carlo method at a concentration of 0.15 M. Each system underwent 5,000 steps of the steepest energy minimization to remove the steric overlap. All the systems were then subjected to an NVT (constant number of particles, Volume, and Temperature) equilibration phase for 125,000 steps. This phase used the V-rescale temperature-coupling method, with a constant coupling of 1 ps at 303.15 K (52). Subsequently, the molecular dynamics was carried out for 100 ns using the CHARMM 36 m force field (53). Gromacs utilities were used to evaluate the root-mean-square deviation (RMSD) of the complexes (as well as that of each protein and ligand), root-mean-square fluctuation (RMSF), hydrogen bonds and radius of gyration, the data were graphed using the GRACE program (XMGRACE, Version 5.1.3).

### 2.12 Statistical analysis

Data are expressed in Mean ± SEM. The differences between the groups were analyzed by parametric two-way ANOVA test with Tukey’s posttest using the Graph Pad Prism software 8, the differences were considered statistically significant at *P* < 0.05. Experiments with Mtb were analyzed *via* a one-way ANOVA test with multiple observations.

## 3 Results

### 3.1 Body and adipose tissue parameters

**Figure 1:**
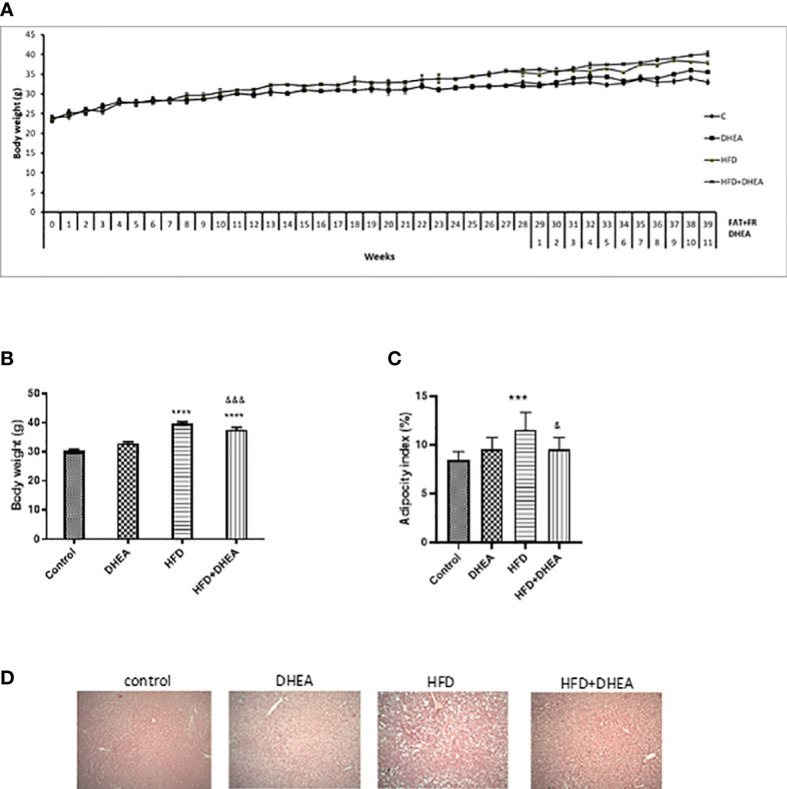
DHEA supplementation effects on body weight, adipocyte tissue, and steatosis induced by HFD. Mice were fed with chow or HFD for 40 weeks and supplemented with or without DHEA (0.05% w/w) starting at week 28. Weekly body weight **(A)**, final body weight **(B)**, and white adiposity index **(C)**. H&E stained livers showing levels of steatosis between groups (100× magnification) **(D)**. Data are means ± SEM (n = 9 per group). ***P < 0.001 ****P < 0.0001 versus C; &P < 0.05, &&&P < 0.001 versus HFD according to one-way ANOVA analysis with multiple comparisons.

At the end of the treatments, HFD and HFD + DHEA groups weighed significantly more than C animals (*p* < 0.0001). The presence of DHEA did not lead to significant differences from their corresponding group (C *vs.* DHEA n.s.). However, DHEA treatment significantly reduced body weight in HFD (*p* < 0.001 HFD + DHEA *vs.* HFD) (**Figures 1A, B** and **Table 1**). Abdominal white adipose tissue was significantly increased in HFD mice with respect to C mice (*p* < 0.0001 *vs.* C). However, DHEA treatment in HFD mice significantly reduced it (*p* < 0.001 HFD + DHEA *vs.* HFD), restoring the white fat weight to control values (**Table 1**). Adiposity index analysis demonstrated that HFD mice exhibited a significant increase in body fat compared with C mice (*p* < 0.001 HFD *vs.* C). The administration of DHEA induced a reduction in the adiposity percentage of the animals under the HFD treatment (*p* < 0.05 HFD *vs.* HFD + DHEA) (**Figure 1C**). H&E-stained livers indicate hepatosteatosis and fatty liver disease induced by HFD with respect to control livers that were partially reversed in HFD mice treated with DHEA (**Figure 1D**).

**Table 1:** Body and metabolic parameters: Mice were fed with chow or HFD for 40 weeks and supplemented with or without DHEA (0.05% w/w) starting at week 28.

	Control	DHEA	HFD	HFD+DHEA
	*Corporal parameters*			
Initial weight (g)	20.4 ± 0.3	20.2 ± 0.	20.0 ± 0.4	20.0 ± 0.2
Final weight (g)	30.3 ± 0.4	33.0 ± 0.5	39.6 ± 0.6^****^	37.6 ± 0.3^****^_&&&_
White adipose tissue (g)	3.7 ± 0.3	2.6 ± 0.1	5.6 ± 0.2^****^	4.2 ± 0.4 _&&&_
	*Serum biochemical parameters*			
TG (mg/dL)	93.0 ± 7.4	63.2 ± 6.2^**^	178.5 ± 13.9^**^	71.7 ± 2.3^*^_&&&_
T-Ch (mg/dl)	158.7 ± 16.7	132.2 ± 9.5	164.2 ± 9.5	187.4 ± 15.7
HDL-Ch	65.5 ± 6.5	87.0 ± 5.2	66.5 ± 6.1	101.1 ± 4.5^***^_&&&_
Fasted glycemia (mg/dl)	123.5 ± 5.3	136.5 ± 8.5	153.4 ± 14.3	140.5 ± 10.4
Fasted insulin (ng/ml)	0.8 ± 0.1	1.1 ± 0.2	1.7 ± 0.5^**^	1.3 ± 0.1
HOMA score	6.1 ± 0.7	10.2 ± 1.6	14.8 ± 2.5 ^**^	8.2 ± 1.4_&_

Body weight was measured along the experiment and statistically analyzed at the final point; the adiposity index is the sum of the total white adipose tissue relative to body weight; biochemical parameters were measured at the end of the treatment. Data are means ± SEM (n = 9). *P < 0.05, **P < 0.01, ***P < 0.001 ****P < 0.0001 versus C; _&_P < 0.05, _&&&_P < 0.001 versus HFD according to one-way ANOVA analysis with multiple comparisons.

### 3.2 Metabolic parameters

Plasma triglycerides concentrations were significantly increased in HFD mice compared with C animals (*p* < 0.01 HFD *vs.* C). DHEA treatment significantly decreased plasma triglycerides (TG) concentrations in the control group (*p* < 0.01 DHEA *vs.* C) and HFD + DHEA group (*p* < 0.05 HFD + DHEA *vs.* C). The presence of DHEA in the HFD + DHEA group significantly reduced the TG related to HFD (*p* < 0.001 HFD+DHEA *vs.* HFD). No significant differences were found in the total plasma cholesterol concentration in either group. However, high-density lipoprotein cholesterol (HDL-Ch) was significantly increased in the plasma of HFD + DHEA animals related to HFD (*p* < 0.001 HFD + DHEA *vs.* HFD) and C mice (*p* < 0.001 HFD + DHEA *vs.* C) (**Table 1**).

There were no significant differences in plasma fasting glucose among the groups (**Table 1**). However, fasting insulin of HFD mice had significant increases compared with C animals (*p* < 0.01 HFD *vs.* C). This increase suggests insulin resistance. Even though HFD + DHEA mice had a tendency to undergo a decrease in insulin compared with HFD animals, this was not significant. The HOMA score of the HFD mice was significantly higher than that of the C mice (*p* < 0.01 HFD *vs.* C). DHEA decreased the score in the HFD + DHEA group with respect to the HFD group (*p* < 0.05 HFD+DHEA *vs.* HFD) (**Table 1**).

**Figure 2:**
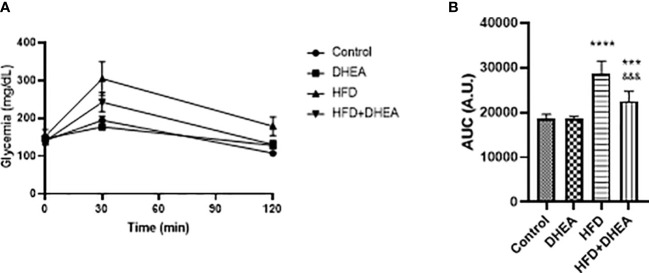
Effects of DHEA supplementation on glucose intolerance induced by HFD. Glucose tolerance test **(A)** and area under the curve **(B)**. Mice were fasted for 12h and oral glucose solution (80% w/v) was administrated at a dose of 2 g/kg body weight. Data are means of plasma glucose levels (mg/dl) ± SEM (*n* = 9). ***P < 0.001, ****P < 0.0001 *versus* C and ^&&&^ *P* < 0.001 versus HFD according to one-way ANOVA analysis with multiple comparisons.

At the end of the treatment, an oral glucose tolerance test (OGTT) was performed to evaluate insulin sensitivity. Plasma glucose levels after 30 min were significantly increased in the HFD and HFD + DHEA groups. Only glycemia in the HFD group remained elevated at the end of the test (**Figure 2A**). The analysis of the area under the curve (AUC) demonstrated a significant increase in HFD glucose levels (*p* < 0.0001 HFD *vs.* C), which was partially reversed by DHEA treatment (*p* < 0.001 HFD + DHEA *vs.* HFD). Glucose level remained significantly elevated with respect to the control group (*p* < 0.001 HFD + DHEA *vs.* C) (**Figure 2B**) in accordance to HOMA results (**Table 1**). These data suggest that HFD is implicated with the induction of MS and NAFLD that could be partially reversed by DHEA.

### 3.3 Expression of inflammatory parameters in the lung of mice with MS

**Figure 3:**
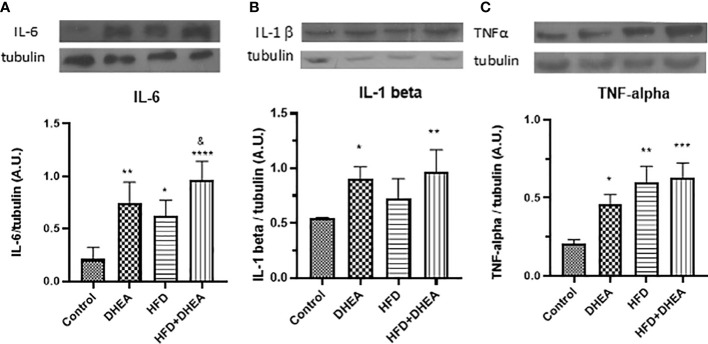
Expression of inflammation mediators. Protein levels of IL-6 **(A)**, IL-1β **(B)**, and TNF-α **(C)** were determined by Western blot in lung homogenates. Representative immunoblots of three experiments in duplicate and graphical representations of protein expressions normalized to β-tubulin are shown. *p < 0.05, **p < 0.01, ***p < 0.001 ****p < 0.0001 *versus* C and ^&^ *p* < 0.05 *versus* HFD.

Exacerbated alterations in the pulmonary immune response related to obesity have been associated with the alteration of metabolically activated macrophages (54), where pro-inflammatory M1 macrophages induce aerobic glycolysis (55). With the aim to explore DHEA’s potential to modulate inflammatory misbalance in the context of obesity, we analyzed expression levels of pro-inflammatory cytokines in the lungs of mice. Once MS and glucose intolerance were established, and after DHEA treatment, animals were euthanized. We analyzed the inflammatory profile in the lungs by evaluating the cytokine expression. The expression of IL-6 was increased in HFD mice (*p* < 0.05 HFD *vs.* C) and in DHEA mice with respect to C mice (*p* < 0.01 DHEA *vs.* C). The presence of DHEA in HFD increases this tendency even more with respect to C group (*p*<0.0001 HFD + DHEA *vs.* C) and respect to HFD mice (*P* < 0.05 HFD + DHEA *vs.* HFD) (**Figure 3A**). Similarly, IL-1β expression showed a tendency to increase in the HFD animals compared with C animals, although the increase was not significant. However, the presence of DHEA significantly increased the expression of IL-1β in the DHEA group (*p* < 0.05 DHEA *vs.* C) and HFD + DHEA group (*p* < 0.01 HFD + DHEA *vs.* C) (**Figure 3B**). TNFα expression also significantly increased in animals under HFD treatment (*p* < 0.01 HFD *vs.* C); the presence of DHEA reinforced this response (*p* < 0.001 HFD + DHEA *vs.* C) (**Figure 3C**).

**Figure 4:**
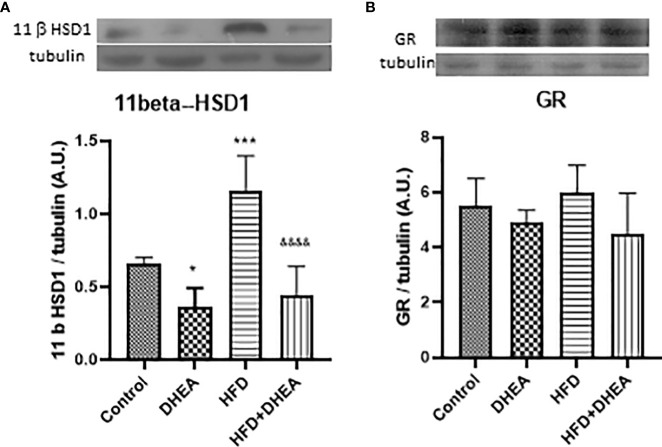
Expression of 11β-HSD 1 and GR. Expressions of 11β-HSD 1 **(A)** and GR **(B)** were measured by Western blot in lung homogenates. Representative immunoblots of three experiments in duplicate and graphical representations of protein expressions normalized to the amount of β-tubulin are shown. *p < 0.05 ***p < 0.001 *versus* C and ^&&&&^ *p* < 0.0001 *versus* HFD.

11β-HSD1 is expressed in the liver and lungs; it is induced in adipose tissue of obese humans and rodent obesity models (11). There is evidence that 11β-HSD1 is up-regulated by pro-inflammatory cytokines such as IL-1β and TNF-α in adipocytes and macrophages (56, 57). Because local levels of corticosterone increased after the induction of immune lung cells (58), we explored the expression of 11β-HSD1 in the lungs after the treatments. The HFD diet significantly increased 11β-HSD1 expression (*p* < 0.001 HFD *vs.* C); DHEA down-regulates this effect in HFD + DHEA mice compared with HFD group (*p* < 0.0001 HFD + DHEA *vs.* HFD) (**Figure 4A**). GC receptor expression did not vary with treatments (**Figure 4B**), which suggests that the modulation of inflammatory response in the lung could be due to GC intracellular activation.

### 3.4 Modulation of macrophage microbicide capacity by DHEA

**Figure 5:**
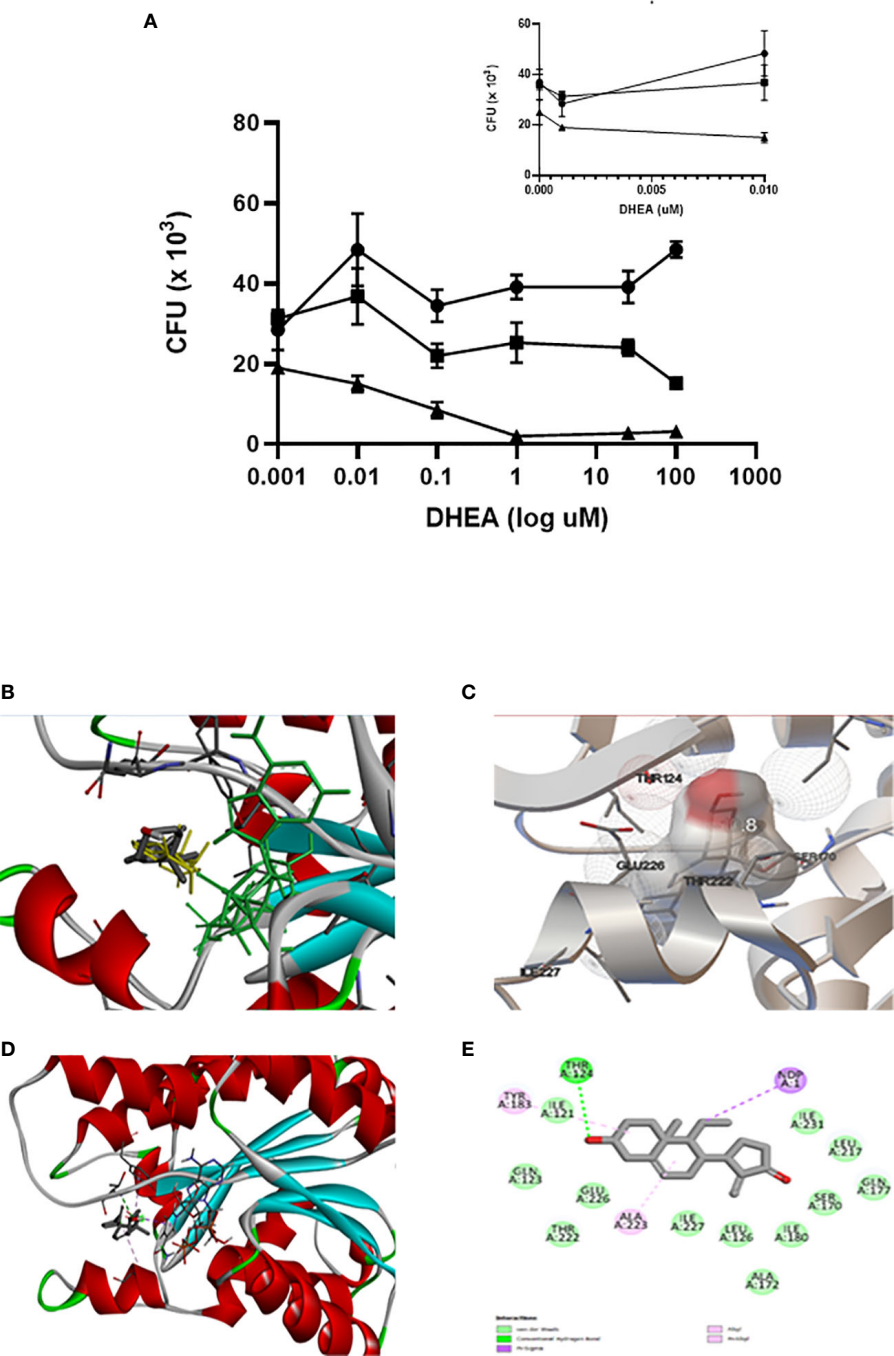
DHEA interacts with 11β-HSD 1 occupying the active site as its natural substrate corticosterone. The effect of DHEA on the killing of Mtb by macrophages is DHEA dose-dependent. Incubations time of macrophages with DHEA. (-●- T = 1, -■- T = 2, -▲- T = 3 days). The insert in the arithmetic scale shows UFC in the absence and presence of the lowest DHEA concentrations **(A)**. Superimposed analysis by docking between DHEA (gray) and 11β-HSD1 co-crystalized with corticosterone (yellow) shows that both molecules share the same pose into the binding site of 11β-HSD1 **(B)**. Rigid docking simulations performed by Autodock Vina evidence that DHEA interacts with the active site of 11β-HSD1 establishing a hydrogen bond **(C)**. Visualization of the active site in the presence of NADH cofactor docked with DHEA **(D)**. DHEA into the active site of 11β-HSD1 establishes a hydrogen bond with Thr124 of the enzyme **(E)**.

The results in the present study indicate that mice with MS have increased expression of 11β-HSD1 in the lungs. It was down-regulated by DHEA treatment, which suggests that local inhibition of corticosterone activation could promote cytokine synthesis. In previous work, we have demonstrated that mice with T2DM and TB are more prone to lung disease than TB mice. Br-DHEA treatment lowered lung bacillary load and pneumonia, which decreased circulating corticosterone and 11β-HSD1 expression (59). Alveolar macrophages and epithelial cells are the first line of defense against bacterial growth, because they initiate intracellular killing and antigen presentation (60); they also express 11β-HSD1 (61). In humans, DHEA competes with cortisone for binding to 11β-HSD1, which impairs the activation of cortisone to cortisol (62). For this reason, we explored the microbicide activity of alveolar macrophages (THP-1) infected with Mtb H37Rv (MOI 5:1) in the absence or in the presence of various concentrations of DHEA (from 0.001 to 100 μM) incubated for cero, 1 or 2 days. The presence of DHEA dose-response inhibited the viability of Mtb within the macrophages as demonstrated by the CFUs’ growth after macrophages’ lyses. On day 1 of incubation, DHEA 0.1 μM temporarily inhibited the mycobacteria growth. DHEA 0.1 μM or higher was enough to significantly reduce the bacilli growth after two days of incubation. For DHEA 1 μM, there was almost complete inhibition of mycobacteria survival after that 2-day period. Negative controls are included in an arithmetic scale with the lowest doses of DHEA (**Figure 5A**). DHEA did not have any observable toxic effects on macrophages. The microbicide activity could be attributed to a direct effect on 11β-HSD1 activity (62), an indirect action due that DHEA promoting macrophage autophagy (41), or both.

The molecular docking strategy is a practical tool for rational drug design, because it predicts the binding of a ligand to a protein in a stable conformation (63). The enzyme 11β-HSD1 is highly expressed in mouse bronchial epithelial cells (64), where it has a preference for the reduction of 7-keto-DHEA to 7β-hydroxyl-DHEA (62, 65). Overlaying through the docking of DHEA with 11β-HSD1 co-crystallized with corticosterone (PDB ID 1Y5R) confirmed that DHEA superimposed well with corticosterone into the catalytic site of 11β-HSD1 (**Figure 5B**), Molecular docking by means of Autodock Vina allowed us to evaluate the binding interactions and energy scores of DHEA in the target enzyme 11β-HSD1 in the presence of the cofactor NADPH (energy score = −10.5 kcal/mol). We visualized the best pose in a hole molecule (**Figure 5C**). Discovery Studio software was used to identify the hydrogen bond between DHEA and Thr124 within the catalytic site of the molecule (**Figures 5D, E**). This results suggests that DHEA could displace corticosterone activation of 11β-HSD1 promoting pro-inflammatory state in the lung.

### 3.5 Microbicide effect of DHEA through interaction with the active site of InhA

**Figure 6:**
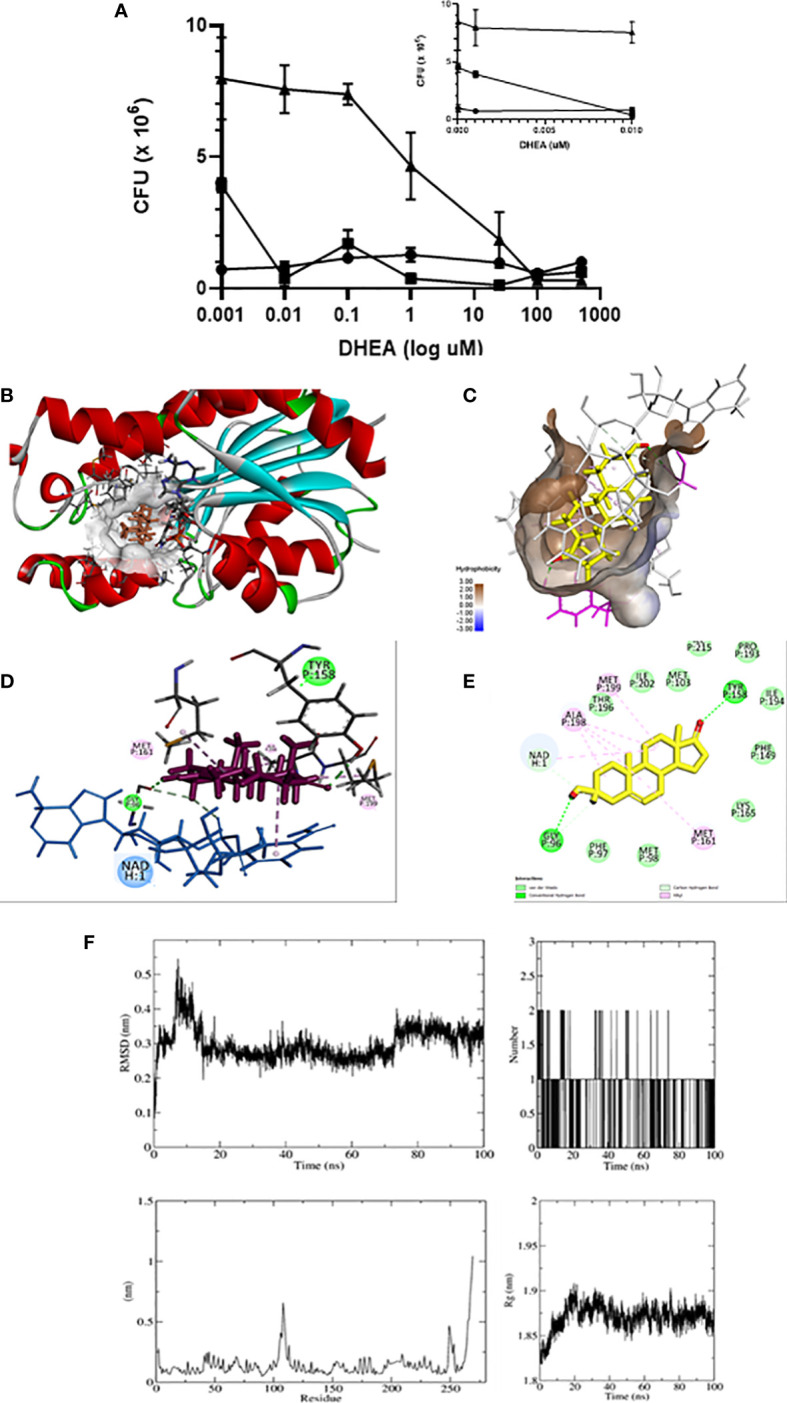
DHEA has microbicide effect on Mtb through interaction with InhA. Anti-mycobacterial activity of DHEA was evaluated by CFUs using the minimum inhibitory concentration (MIC) assay. Incubations time of Mtb with DHEA. (-●- T = 1, -■- T = 2, -▲- T = 3 days). The insert in the arithmetic scale shows UFC in the absence and presence of the lowest DHEA concentrations **(A)**. Molecular docking shows that DHEA interacts with InhA enzyme of Mtb in the active site in the presence of cofactor NADH **(B)**. The hydrophobic pocket is shown **(C)**. The 3D representations **(D)** and 2D diagram **(E)** of InhA interacting with DHEA display its interactions by two hydrogen bonds with Tyr 158 and Gly95 in the active site. Molecular dynamic (MD) simulation studies showing the stability of the protein-ligand complex between InhA and DHEA: RMSD plot, Radius of gyration (Rg) plot, RMSF plot, and hydrogen bonds throughout the simulation **(F)**.

Because mammalian 11β-HSD1 and mycobacterium InhA belongs to the same SDR family, it could be possible that DHEA inhibits InhA as it was described to the enzyme. Next, we tested whether DHEA had a direct microbicide effect against mycobacteria. The test included counting CFUs of bacilli incubated in the absence or presence of various concentrations of DHEA (0.001–500 μM). **Figure 6A** displays the time and dose-dependent microbicide effect of DHEA. After 2 days of incubation of DHEA, doses of 0.01 μM or higher were sufficient for inhibiting bacilli growth. After 3 days, the minimum effective dose was 1 μM. Negative controls are included in the arithmetic scale with the lowest doses of DHEA. These results strongly suggest that DHEA could have a microbicide effect on Mtb.

The short chain dehydrogenase-reductase (SDR) comprises a huge family of proteins (from bacteria to mammalian), with NADH or NADPH as a coenzyme. Considering that mammalian 11β-HSD1 and bacterial InhA are members of the SDR family, we explored *in silico* to see if the microbicide effect of DHEA on Mtb survival could be related to an inhibition of the InhA enzyme. Using Autodock Vina, the inspection of the docking poses revealed the ability of DHEA to fit preferentially in the active site of the InhA enzyme (energy score = −8.3 kcal/mol) (PDB ID 4TRN) (**Figure 6B**). Worthy of note, it has been established several hydrophobic interactions (Van der Walls and alkyl) with non-polar residues lining a hydrophobic pocket (**Figure 6C**). The Discovery Studio software was used to identify the hydrogen bond between carbonyl oxygen of DHEA and the hydroxyl group of catalytic Tyr158 into the active site of InhA (**Figure 6D**). The 2D diagrams for interactions (**Figure 6E**), and the 3D illustrations of the docking pose between DHEA and InhA active site reveal that DHEA had a binding affinity by the active site of the InhA.

Molecular dynamics is considered a validation method of interaction studies because allows the evaluation of the stability of the protein-ligand complex obtained from coupling studies (66). In this work, the interaction of DHEA with InhA per 100 ns was evaluated. The root mean square deviation (RMSD) reveals information about the structural stability of the system by measuring the average change in the displacement of a group of atoms with respect to a reference frame (67). The variation was between 0.1 and 0.55 nm, the movement of the complex is considered stable with the greatest fluctuation at 10 ns, and there were no relevant changes with respect to the results found by coupling molecular. The molecular dynamics start with two DHEA hydrogen bonds, eventually a third is formed, but only one remains stable throughout the entire trajectory. The root mean square fluctuation (RMSF) results for a given ligand reflect the fluctuations of the loops during the simulation period. It was observed that there are two regions with greater fluctuation, one between residue 150 and 120, and 250. These two regions correspond to the response of the interaction with DHEA. The radius of gyration (Rg) is a measure of the compactness of the protein during the simulation. The values obtained are between 1.82 and 1.91, which represents a low rate of variation in the degree of compaction of the protein, indicating that the protein maintains as stable structure when interacting with DHEA (**Figure 6F**). All the results indicate that the ligand-protein interaction between DHEA and InhA is stable.

## 4 Discussion

The vertiginous increase worldwide in cases of T2DM and TB is even more frequent in low or middle-income countries and aggravated by intense migratory flows. The prevalence leads to the assumption that individuals with both diseases will continue to increase in the coming decades (27). Furthermore, diabetic patients have a tripled risk of contracting TB and having inadequate response to treatment. It is estimated that diabetes increases the probability of death from TB in patients not infected with HIV by more than 10% (68). People with TB-DM had more severe TB presentations, increased risk of treatment failure (69), TB relapse, and delayed sputum culture conversion (70). In the search for associations between both pathologies, it has been suggested that diabetes weakens the immune system. A weakened immune system predisposes patients to the proliferation of *Mycobacterium tuberculosis* and/or progression to symptomatic disease.

Patients with TB have a reduced DHEA/cortisol ratio (31) (71). It has also been proposed that DHEA treatment could aid in resolving TB infection by promoting the granuloma formation and Th1 cytokine pattern (34, 41). The essential components in the control of bacterial growth are alveolar macrophages activated by interleukin 12 (IL-12) and interferon gamma produced by Th1 cells. Therefore, macrophages are the first line of the defense against mycobacterium. The defense includes phagocytosis, the killing by concerted action between free radicals and lysosomal enzymes, and antigen presentation to lymphocytes (60). In addition, macrophages express 11β-HSD1 (61), which have high activity that could be regulated by DHEA (25). The effectiveness of DHEA as coadjutant therapy for the treatment of T2DM/TB comorbidity was previously explored (59). However, the effect of DHEA on macrophage activity against Mtb and the direct microbicide activity of DHEA have not been molecularly explored.

In the last decade, numerous *in silico* analyses have validated new hypotheses for druggable targets. This validation established a comprehensive platform technology for drug discovery. Among druggable targets against mycobacteria that meet the requirements for bacteria survival, the cell wall synthesis is one of the most attractive, because it contributes to support and protection (72). The Mtb cell wall is composed of three covalently linked components: long-chain mycolic acids, a cross-linked network of peptide-glycan, and highly branched arabinogalactan that establishes a promise target for the development of more effective anti-TB molecules (73). InhA is a key enzyme in the metabolism and synthesis of the Mtb cell wall and has been clinically validated by the prodrugs isoniazid (INH) and ethionamide. However, the emergence of INH resistance is considered a global threat. New molecules must be generated to identify new direct inhibitors of InhA (74-76).

The experimental model of MS in this study demonstrates that mice treated high-fat diet and DHEA have a better metabolic profile, an increase in the expression of pro-inflammatory cytokines and a decrease in the enzyme 11β-HSD1, which is key in the local activation of corticosterone. Thus, DHEA could control the anti-inflammatory effect of GC in the context of TB and T2DM comorbidity. The enzyme InhA from *Mycobacterium tuberculosis* and the enzyme 11β-HSD1 from mammals belong to the same SDR family, and DHEA is a competitive inhibitor of 11β-HSD1. These two factors contributed to the possibility of exploring whether DHEA could be a potential inhibitor of the InhA.

The homologies found, between mammalian 11β-HSD1, that activate GC in the lung and the reductase InhA involved in the synthesis of mycolic acid provide a research pathway for exploring whether the inhibitors described for 11β-HSD1 would have any effect on the bacterial enzyme InhA. If so, we hypothesize that DHEA could promote the stimulation of the host’s response to infection, along with the inhibition of bacterial growth; this process could improve the resolution of TB. For this purpose, we first evaluated *in vivo* whether DHEA treatment could modulate lung inflammation induced in an experimental model of MS. Due to macrophages expressing 11β-HSD1 (77) and DHEA inhibiting the enzyme activity (24, 65), we evaluated *in vitro* the effects of DHEA on the phagocytic capacity of alveolar macrophages challenged with Mtb H37Rv. Based on the structural homology between 11β-HSD1 and InhA (35) we also investigated *in vitro* the direct effect of DHEA on mycobacteria viability and explored *in silico* whether if InhA enzyme of Mtb could interact with DHEA similar to the way mammalian 11β-HSD1 interacts with it. Using docking analysis and molecular dynamics, we could demonstrate that DHEA binds with the catalytic pocket of InhA.

InhA has an active site where catalytic residues Lys165 and Tyr158 are located in the substrate-binding pocket interacting with the long-chain fatty acyl substrates required for the synthesis of mycolic acid (75, 78). INH is a prodrug that is oxidized by KatG creating the INH-NAD adduct, a competent InhA inhibitor (76). Because direct inhibitors bind to InhA without requiring prior activation by KatG, it would be worth exploring novel agents to be used in future anti-TB treatments. To the best of our knowledge, this is the first report about the hydrogen bonding interaction between the hydroxyl group of Tyr158 of InhA and the oxygen atom of the carbonyl group of DHEA suggesting further research about DHEA analogs to overcome the drug resistance or synergize the current treatments. Testing the inhibitory efficacy on Mtb multi-drug resistance (MDR) and extensively drug resistance (XDR) strains, together with biochemical studies with the purified enzyme and its resistance mutants are pending matters.

Our results suggest that the administration of DHEA can elevate the concentration of pro-inflammatory cytokine in the lung through the inhibition of GC activation by 11β-HSD1. DHEA administration can also simultaneously inhibit mycobacterial InhA. The microbicide effect of DHEA through interaction with InhA suggests a rational drug design of novel inhibitors of InhA as adjuvant therapy. These results provide molecular support to our previous reports in the BALB/C mouse TB model. In this previous report, a new water-miscible formulation of Br-DHEA resulted in lowered pneumonia and, greater and, fast bacterial clearance, along with a reduction of 11β-HSD1 activity and corticosterone production that effectively reactivates the protective anti-TB immunity (59,79).

## Data availability statement

The datasets presented in this study can be found in online repositories. The names of the repository/repositories and accession number(s) can be found in the article/supplementary material.

## Ethics statement

The animal study was reviewed and approved by Institutional Council for Care and Use of Experimental Animals (CICUAL), School of Pharmacy and Biochemistry, University of Buenos Aries.

## Author contributions

IH-B and YS-P performed, organized, and analyzed the results. AR-C performed the bioinformatic analysis. EB, DM-E, BM-C, and JB-P contributed to the supervision of the experimental work. IT-V and AT advised on the diet high in fats and carbohydrates. AC and RH-P contributed to the background work, conceived the experiments, and wrote the manuscript. All authors contributed to the article and approved the submitted version.

## References

[1] Eckel RH Grundy SM Zimmet PZ. The metabolic syndrome. Lancet (2010) 375(9710):181–3. doi: 10.1016/S0140-6736(09)61794-3 20109902

[2] Reaven GM. Banting lecture 1988. role of insulin resistance in human disease. Diabetes (1988) 37(12):1595–607. doi: 10.1056/NEJM199602083340607 3056758

[3] Halpern A Mancini MC Magalhães MEC Fisberg M Radominski R Bertolami MC. Metabolic syndrome, dyslipidemia, hypertension and type 2 diabetes in youth: From diagnosis to treatment. Diabetol Metab Syndr (2010) 2:55. doi: 10.1186/1758-5996-2-55 20718958 PMC2939537

[4] Sutherland JP McKinley B Eckel RH. The metabolic syndrome and inflammation. Metab Syndr Relat Disord (2004) 2(2):82–104. doi: 10.1089/met.2004.2.82 18370640

[5] Harford KA Reynolds CM McGillicuddy FC Roche HM. Fats, inflammation and insulin resistance: Insights to the role of macrophage and T-cell accumulation in adipose tissue. Proc Nutr Soc (2011) 70(4):408–17. doi: 10.1017/S0029665111000565 21835098

[6] Coppack SW. Pro-inflammatory cytokines and adipose tissue. Proc Nutr Soc (2001) 60(3):349–56. doi: 10.1079/pns2001110 11681809

[7] Xu H Barnes GT Yang Q Tan G Yang D Chou CJ. Chronic inflammation in fat plays a crucial role in the development of obesity-related insulin resistance. J Clin Invest (2003) 112(12):1821–30. doi: 10.1172/JCI19451 PMC296998 14679177

[8] Zammit AR Katz MJ Derby C Bitzer M Lipton RB. Chronic kidney disease in non-diabetic older adults: Associated roles of the metabolic syndrome, inflammation, and insulin resistance. PloS One (2015) 10(10):e0139369. doi: 10.1371/journal.pone.0139369 26431218 PMC4592063

[9] Jia G Aroor AR Martinez-Lemus LA Sowers JR. Overnutrition, mTOR signaling, and cardiovascular diseases. Am J Physiol Regul Integr Comp Physiol (2014) 307(10):R1198–206. doi: 10.1152/ajpregu.00262.2014 PMC4233289 25253086

[10] Rajan V Chapman KE Lyons V Jamieson P Mullins JJ Edwards CRW. Cloning, sequencing and tissue-distribution of mouse 11β-hydroxysteroid dehydrogenase-1 cDNA. J Steroid Biochem Mol Biol (1995) 52(2):141–7. doi: 10.1016/0960-0760(94)00159-j 7873449

[11] Masuzaki H Paterson J Shinyama H Morton NM Mullins JJ Seckl JR. A transgenic model of visceral obesity and the metabolic syndrome. Science (2001) 294(5549):2166–70. doi: 10.1126/science.1066285 11739957

[12] Tomlinson JW Walker EA Bujalska IJ Draper N Lavery GG Cooper MS. 11β-hydroxysteroid dehydrogenase type 1: A tissue-specific regulator of glucocorticoid response. Endocr Rev (2004) 25(5):831–66. doi: 10.1210/er.2003-0031 15466942

[13] Draper N Stewart PM. 11 -hydroxysteroid dehydrogenase and the pre-receptor regulation of corticosteroid hormone action. J Endocrinol (2005) 186(2):251–71. doi: 10.1677/joe.1.06019 16079253

[14] Chapman K Holmes M Seckl J. 11β-hydroxysteroid dehydrogenases: Intracellular gate-keepers of tissue glucocorticoid action. Physiol Rev (2013) 93(3):1139–206. doi: 10.1152/physrev.00020.2012 PMC3962546 23899562

[15] Zhou HY Hu GX Lian QQ Morris D Ge RS. The metabolism of steroids, toxins and drugs by 11β-hydroxysteroid dehydrogenase 1. Toxicology (2012) 292(1):1–12. doi: 10.1016/j.tox.2011.11.012 22154985

[16] Atanasov AG Nashev LG Schweizer RA Frick C Odermatt A. Hexose-6-phosphate dehydrogenase determines the reaction direction of 11beta-hydroxysteroid dehydrogenase type 1 as an oxoreductase. FEBS Lett (2004) 571(1-3):129–33. doi: 10.1016/j.febslet.2004.06.065 15280030

[17] Prince PD Santander YA Gerez EM Höcht C Polizio AH Mayer MA. Fructose increases corticosterone production in association with NADPH metabolism alterations in rat epididymal white adipose tissue. J Nutr Biochem (2017) 46:109–16. doi: 10.1016/j.jnutbio.2017.02.021 28499147

[18] Morton NM. Obesity and corticosteroids: 11β-hydroxysteroid type 1 as a cause and therapeutic target in metabolic disease. Mol Cell Endocrinol (2010) 316(2):154–64. doi: 10.1016/j.mce.2009.09.024 19804814

[19] Anagnostis P Katsiki N Adamidou F Athyros VG Karagiannis A Kita M. 11beta-hydroxysteroid dehydrogenase type 1 inhibitors: Novel agents for the treatment of metabolic syndrome and obesity-related disorders? Metabolism (2013) 62(1):21–33. doi: 10.1016/j.metabol.2012.05.002 22652056

[20] Rahman SM Janssen RC Choudhury M Baquero KC Aikens RM de la Houssaye BA. CCAAT/enhancer-binding protein β (C/EBPβ) expression regulates dietary-induced inflammation in macrophages and adipose tissue in mice. J Biol Chem (2012) 287(41):34349–60. doi: 10.1074/jbc.M112.410613 PMC3464541 22902781

[21] Yang Z Zhu P Guo C Zhu X Sun K. Expression of 11β-hydroxysteroid dehydrogenase type 1 in human fetal lung and regulation of its expression by interleukin-1β and cortisol. J Clin Endocrinol Metab (2009) 94(1):306–13. doi: 10.1210/jc.2008-1534 18840637

[22] Cain DW Cidlowski JA. Immune regulation by glucocorticoids. Nat Rev Immunol (2017) 17(4):233–47. doi: 10.1038/nri.2017.1 PMC9761406 28192415

[23] André DM Calixto MC Sollon C Alexandre EC Tavares EBG Naime ACA. High-fat diet-induced obesity impairs insulin signaling in lungs of allergen-challenged mice: Improvement by resveratrol. Sci Rep (2017) 7(1):17296. doi: 10.1038/s41598-017-17558-w 29229986 PMC5725490

[24] McNelis JC Manolopoulos KN Gathercole LL Bujalska IJ Stewart PM Tomlinson JW. Dehydroepiandrosterone exerts antiglucocorticoid action on human preadipocyte proliferation, differentiation, and glucose uptake. Am J Physiol Endocrinol Metab (2013) 305(9):E1134–44. doi: 10.1152/ajpendo.00314.2012 PMC3840204 24022868

[25] Apostolova G Schweizer RAS Balazs Z Kostadinova RM Odermatt A. Dehydroepiandrosterone inhibits the amplification of glucocorticoid action in adipose tissue. Am J Physiol Endocrinol Metab (2005) 288(5):E957–64. doi: 10.1152/ajpendo.00442.2004 15613680

[26] Furin J Cox H Pai M. Tuberculosis. Lancet (2019) 393(10181):1642–56. doi: 10.1016/S0140-6736(19)30308-3 30904262

[27] Dooley KE Chaisson RE. Tuberculosis and diabetes mellitus: Convergence of two epidemics. Lancet Infect Dis (2009) 9(12):737–46. doi: 10.1016/S1473-3099(09)70282-8 PMC2945809 19926034

[28] Jeon CY Murray MB. Diabetes mellitus increases the risk of active tuberculosis: A systematic review of 13 observational studies. PloS Med (2008) 5(7):e152. doi: 10.1371/journal.pmed.0050152 18630984 PMC2459204

[29] Baker RW Walker BR Shaw RJ Honour JW Jessop DS Lightman SL. Increased cortisol: Cortisone ratio in acute pulmonary tuberculosis. Am J Respir Crit Care Med (2000) 162(5):1641–7. doi: 10.1164/ajrccm.162.5.9912119 11069789

[30] Abbott AN Welsh KJ Hwang S-A Płoszaj P Choudhury T Boyd S. IL-6 mediates 11βHSD type 2 to effect progression of the mycobacterial cord factor trehalose 6,6’-dimycolate-induced granulomatous response. Neuroimmunomodulation (2011) 18(4):212–25. doi: 10.1159/000323776 PMC3068753 21389736

[31] Rook GA Hernandez-Pando R. Pathogenetic role, in human and murine tuberculosis, of changes in the peripheral metabolism of glucocorticoids and antiglucocorticoids. Psychoneuroendocrinology (1997) 22(Suppl 1):S109–13. doi: 10.1016/s0306-4530(97)00014-0 9264156

[32] Fernández RDV Díaz A Bongiovanni B Gallucci G Bértola D Gardeñez W. Evidence for a more disrupted immune-endocrine relation and cortisol immunologic influences in the context of tuberculosis and type 2 diabetes comorbidity. Front Endocrinol (Lausanne) (2020) 11:126. doi: 10.3389/fendo.2020.00126 32265833 PMC7099637

[33] Rook GA Hernandez-Pando R Lightman SL. Hormones, peripherally activated prohormones and regulation of the Th1/Th2 balance. Immunol Today (1994) 15(7):301–3. doi: 10.1016/0167-5699(94)90075-2 8086097

[34] Hernandez-Pando R de la Luz Streber M Orozco H Arriaga K Pavon L Al-Nakhli SA. The effects of androstenediol and dehydroepiandrosterone on the course and cytokine profile of tuberculosis in BALB/c mice. Immunology (1998) 95(2):234–41. doi: 10.1046/j.1365-2567.1998.00601.x PMC1364310 9824481

[35] Jörnvall H Krook M Persson B Atrian S Gonzàlez-Duarte R Jeffery J. Short-chain Dehydrogenases/Reductases (SDR). Biochemistry (1995) 34(18):6003–13. doi: 10.1021/bi00018a001 7742302

[36] Baker ME. Enoyl-acyl-carrier-protein reductase and mycobacterium tuberculosis InhA do not conserve the tyr-Xaa-Xaa-Xaa-Lys motif in mammalian 11β- and 17β-hydroxysteroid dehydrogenases and drosophila alcohol dehydrogenase [1]. Biochem J (1995) 309(Pt 3):1029–30. doi: 10.1042/bj3091029 PMC1135734 7639680

[37] Banerjee A Dubnau E Quemard A Balasubramanian V Um KS Wilson T. inhA, a gene encoding a target for isoniazid and ethionamide in mycobacterium tuberculosis. Science (1994) 263(5144):227–30. doi: 10.1126/science.8284673 8284673

[38] Leal-Díaz AM Noriega LG Torre-Villalvazo I Torres N Alemán-Escondrillas G López-Romero P. Aguamiel concentrate from agave salmiana and its extracted saponins attenuated obesity and hepatic steatosis and increased akkermansia muciniphila in C57BL6 mice. Sci Rep (2016) 6:34242. doi: 10.1038/srep34242 27678062 PMC5039706

[39] Ibrahim SM El-Denshary ES Abdallah DM. Geraniol, alone and in combination with pioglitazone, ameliorates fructose-induced metabolic syndrome in rats *via* the modulation of both inflammatory and oxidative stress status. PloS One (2015) 10(2):e0117516. doi: 10.1371/journal.pone.0117516 25679220 PMC4332632

[40] Aoki K Tajima K Taguri M Terauchi Y. Effect of dehydroepiandrosterone (DHEA) on akt and protein kinase c zeta (PKCζ) phosphorylation in different tissues of C57BL6, insulin receptor substrate (IRS)1(-/-), and IRS2(-/-) male mice fed a high-fat diet. J Steroid Biochem Mol Biol (2016) 159:110–20. doi: 10.1016/j.jsbmb.2016.03.011 26976654

[41] Bongiovanni B Mata-Espinosa D D’Attilio L Leon-Contreras JC Marquez-Velasco R Bottasso O. Effect of cortisol and/or DHEA on THP1-derived macrophages infected with mycobacterium tuberculosis. Tuberculosis (2015) 95(5):562–9. doi: 10.1016/j.tube.2015.05.011 26099547

[42] Rodríguez-Flores EM Mata-Espinosa D Barrios-Payan J Marquina-Castillo B Castañón-Arreola M Hernández-Pando R. A significant therapeutic effect of silymarin administered alone, or in combination with chemotherapy, in experimental pulmonary tuberculosis caused by drug-sensitive or drugresistant strains: *In vitro* and *in vivo* studies. PloS One (2019) 14(5):e0217457. doi: 10.1371/journal.pone.0217457 31145751 PMC6542514

[43] Hanwell MD Curtis DE Lonie DC Vandermeerschd T Zurek E Hutchison GR. Avogadro: an advanced semantic chemical editor, visualization, and analysis platform. J Cheminform (2012) 4(1):17. doi: 10.1186/1758-2946-4-17 22889332 PMC3542060

[44] Pettersen EF Goddard TD Huang CC Meng EC Couch GS Croll TI. UCSF ChimeraX: Structure visualization for researchers, educators, and developers. Protein Sci (2021) 30(1):70–82. doi: 10.1002/pro.3943 32881101 PMC7737788

[45] Trott O Olson AJ. AutoDock vina: Improving the speed and accuracy of docking with a new scoring function, efficient optimization, and multithreading. J Comput Chem (2010) 31(2):455–61. doi: 10.1002/jcc.21334 PMC3041641 19499576

[46] Morris GM Ruth H Lindstrom W Sanner MF Belew RK Goodsell DS. AutoDock4 and AutoDockTools4: Automated docking with selective receptor flexibility. J Comput Chem (2009) 30(16):2785–91. doi: 10.1002/jcc.21256 PMC2760638 19399780

[47] Abraham MJ Murtola T Schulz R Páll S Smith JC Hess B. Gromacs: High performance molecular simulations through multi-level parallelism from laptops to supercomputers. SoftwareX (2015), 1–2:19–25. doi: 10.1016/j.softx.2015.06.001

[48] Jo S Cheng X Islam SM Huang L Rui H Zhu A. CHARMM-GUI PDB manipulator for advanced modeling and simulations of proteins containing nonstandard residues. Adv Protein Chem Struct Biol (2014) 96:235–65. doi: 10.1016/bs.apcsb.2014.06.002 PMC4739825 25443960

[49] O'Boyle NM Banck M CA J Morley C Vandermeersch T Hutchison GR. Open babel: An open chemical toolbox. J Cheminform (2011) 3:33. doi: 10.1186/1758-2946-3-33 21982300 PMC3198950

[50] Kim S Lee J Jo S Brooks CL 3rd Lee HS Im W. CHARMM-GUI ligand reader and modeler for CHARMM force field generation of small molecules. J Comput Chem (2017) 38(21):1879–86. doi: 10.1002/jcc.24829 PMC5488718 28497616

[51] Lee J Cheng X Swails JM Yeom MS Eastman PK Lemkul JA. CHARMM-GUI input generator for NAMD, GROMACS, AMBER, OpenMM, and CHARMM/OpenMM simulations using the CHARMM36 additive force field. J Chem Theory Comput (2016) 12(1):405–13. doi: 10.1021/acs.jctc.5b00935 PMC4712441 26631602

[52] Bussi G Donadio D Parrinello M. Canonical sampling through velocity rescaling. J Chem Phys (2007) 126(1):014101. doi: 10.1063/1.2408420 17212484

[53] Vanommeslaeghe K Hatcher E Acharya C Kundu S Zhong S Shim J. CHARMM general force field: A force field for drug-like molecules compatible with the CHARMM all-atom additive biological force fields. J Comput Chem (2010) 31(4):671–90. doi: 10.1002/jcc.21367 PMC2888302 19575467

[54] Manicone AM Gong K Johnston LK Giannandrea M. Diet-induced obesity alters myeloid cell populations in naïve and injured lung. Respir Res (2016) 17:24. doi: 10.1186/s12931-016-0341-8 26956558 PMC4782295

[55] Castoldi A Naffah de Souza C Câmara NO Moraes-Vieira PM. The macrophage switch in obesity development. Front Immunol (2016) 6:637. doi: 10.3389/fimmu.2015.00637 26779183 PMC4700258

[56] Tomlinson JW Moore J Cooper MS Bujalska I Shahmanesh M Burt C. Regulation of expression of 11β-hydroxysteroid dehydrogenase type 1 in adipose tissue: Tissue-specific induction by cytokines. Endocrinology (2001) 142(5):1982–9. doi: 10.1210/endo.142.5.8168 11316764

[57] Thieringer R Le Grand CB Carbin L Cai T-Q Wong B Wright SD. 11β-hydroxysteroid dehydrogenase type 1 is induced in human monocytes upon differentiation to macrophages. J Immunol (2001) 167(1):30–5. doi: 10.4049/jimmunol.167.1.30 11418628

[58] Hostettler N Bianchi P Gennari-Moser C Kassahn D Schoonjans K Corazza N. Local glucocorticoid production in the mouse lung is induced by immune cell stimulation. Allergy (2012) 67(2):227–34. doi: 10.1111/j.1398-9995.2011.02749.x 22111694

[59] López-Torres MO Marquina-Castillo B Ramos-Espinosa O Mata-Espinosa D Barrios-Payan JA Baay-Guzman G. 16α-bromoepiandrosterone as a new candidate for experimental diabetes-tuberculosis co-morbidity treatment. Clin Exp Immunol (2021) 205(2):232–45. doi: 10.1111/cei.13603 PMC8274213 33866550

[60] Carranza C Juárez E Torres M Ellner JJ Sada E Schwander SK. Mycobacterium tuberculosis growth control by lung macrophages and CD8 cells from patient contacts. Am J Respir Crit Care Med (2006) 173(2):238–45. doi: 10.1164/rccm.200503-411OC PMC2662991 16210664

[61] Zhang Z Coutinho AE Man TY Kipari TMJ Hadoke PWF Salter DM. Macrophage 11β-HSD-1 deficiency promotes inflammatory angiogenesis. J Endocrinol (2017) 234(3):291–9. doi: 10.1530/JOE-17-0223 PMC5574305 28676523

[62] Nashev LG Chandsawangbhuwana C Balazs Z Atanasov AG Dick B Frey FJ. Hexose-6-phosphate dehydrogenase modulates 11β-hydroxysteroid dehydrogenase type 1-dependent metabolism of 7-keto- and 7β-hydroxy-neurosteroids. PloS One (2007) 2(6):e561. doi: 10.1371/journal.pone.0000561 17593962 PMC1891437

[63] Lengauer T Rarey M. Computational methods for biomolecular docking. Curr Opin Struct Biol (1996) 6(3):402–6. doi: 10.1016/s0959-440x(96)80061-3 8804827

[64] Chen J Mishra R Yu Y McDonald JG Eckert KM Gao L. Decreased 11β-hydroxysteroid dehydrogenase 1 in lungs of steroid receptor coactivator (Src)-1/-2 double-deficient fetal mice is caused by impaired glucocorticoid and cytokine signaling. FASEB J (2020) 34(12):16243–61. doi: 10.1096/fj.202001809R PMC7713510 33070362

[65] Balázs Z Nashev LG Chandsawangbhuwana C Baker ME Odermatt A. Hexose-6-phosphate dehydrogenase modulates the effect of inhibitors and alternative substrates of 11β-hydroxysteroid dehydrogenase 1. Mol Cell Endocrinol (2009) 301(1-2):117–22. doi: 10.1016/j.mce.2008.10.021 19010388

[66] Salmaso V Moro S. Bridging molecular docking to molecular dynamics in exploring ligand-protein recognition process: An overview. Front Pharmacol (2018) 9:923. doi: 10.3389/fphar.2018.00923 30186166 PMC6113859

[67] Hospital A Goñi JR Orozco M Gelpí JL. Molecular dynamics simulations: advances and applications. Adv Appl Bioinform Chem (2015) 8:37–47. doi: 10.2147/AABC.S70333 26604800 PMC4655909

[68] Noubiap JJ Nansseu JR Nyaga UF Nkeck JR Endomba FT Kaze AD. Global prevalence of diabetes in active tuberculosis: A systematic review and meta-analysis of data from 2·3 million patients with tuberculosis. Lancet Glob Health (2019) 7(4):e448–60. doi: 10.1016/S2214-109X(18)30487-X 30819531

[69] Armstrong LR Steve Kammerer J Haddad MB. Diabetes mellitus among adults with tuberculosis in the USA, 2010-2017. Diabetes Res Care (2020) 8(1):e001275. doi: 10.1136/bmjdrc-2020-001275 PMC7342266 32641300

[70] Reis-Santos B Gomes T Locatelli R De Oliveira ER Sanchez MN Horta BL. Treatment outcomes in tuberculosis patients with diabetes: A polytomous analysis using Brazilian surveillance system. PloS One (2014) 9(7):e100082. doi: 10.1371/journal.pone.0100082 25003346 PMC4086729

[71] Santucci N D’Attilio L Kovalevski L Bozza V Besedovsky H del Rey A. A multifaceted analysis of immune-endocrine-metabolic alterations in patients with pulmonary tuberculosis. PloS One (2011) 6(10):e26363. doi: 10.1371/journal.pone.0026363 22022605 PMC3192801

[72] Elsayed ZM Eldehna WM Abdel-Aziz MM El Hassab MA Elkaeed EB Al-Warhi T. Development of novel isatin-nicotinohydrazide hybrids with potent activity against susceptible/resistant mycobacterium tuberculosis and bronchitis causing-bacteria. J Enzyme Inhib Med Chem (2021) 36(1):384–93. doi: 10.1080/14756366.2020.1868450J PMC7801109 33406941

[73] Shaku M Ealand C Kana BD. Cell surface biosynthesis and remodeling pathways in mycobacteria reveal new drug targets. Front Cell Infect Microbiol (2020) 10:603382. doi: 10.3389/fcimb.2020.603382 33282752 PMC7688586

[74] Vilchèze C Wang F Arai M Hazbón MH Colangeli R Kremer L. Transfer of a point mutation in mycobacterium tuberculosis inhA resolves the target of isoniazid. Nat Med (2006) 9):1027–9. doi: 10.1038/nm1466 16906155

[75] Hartkoorn RC Pojer F Read JA Gingell H Neres J Horlacher OP. Pyridomycin bridges the NADH- and substrate-binding pockets of the enoyl reductase InhA. Nat Chem Biol (2014) 10(2):96–8. doi: 10.1038/nchembio.1405 24292073

[76] Prasad MS Bhole RP Khedekar PB Chikhale RV. Mycobacterium enoyl acyl carrier protein reductase (InhA): A key target for antitubercular drug discovery. Bioorg Chem (2021) 115:105242. doi: 10.1016/j.bioorg.2021.105242 34392175

[77] Chapman KE Coutinho AE Zhang Z Kipari T Savill JS Seckl JR. Changing glucocorticoid action: 11β-hydroxysteroid dehydrogenase type 1 in acute and chronic inflammation. J Steroid Biochem Mol Biol (2013) 137:82–92. doi: 10.1016/j.jsbmb.2013.02.002 23435016 PMC3925798

[78] Chetty S Armstrong T Sharma Kharkwal S Drewe WC De Matteis CI Evangelopoulos D. New InhA inhibitors based on expanded triclosan and di-triclosan analogues to develop a new treatment for tuberculosis. Pharm (Basel) (2021) 14(4):361. doi: 10.3390/ph14040361 PMC8070701 33919737

[79] Bongiovanni B Díaz A Santucci N D’Attilio LD Bottasso O Hernández Pando R. The immunoregulatory actions of DHEA in tuberculosis, a tool for therapeutic intervention? Front Endocrinol (Lausanne) (2022) 13:892270. doi: 10.3389/fendo.2022.892270 35733782 PMC9207529

